# Mustard Gas Induced Corneal Injury Involves Ferroptosis and p38 MAPK Signaling

**DOI:** 10.1167/iovs.66.1.23

**Published:** 2025-01-10

**Authors:** Nishant R. Sinha, Alexandria C. Hofmann, Laila A. Suleiman, Maxwell T. Jeffrey, William C. Jeffrey, Rajnish Kumar, Ratnakar Tripathi, Rajiv R. Mohan

**Affiliations:** 1 Harry S. Truman Memorial Veterans’ Hospital, Columbia, Missouri, United States; 2 Departments of Veterinary Medicine & Surgery and Biomedical Sciences, University of Missouri, Columbia, Missouri, United States; 3 Mason Eye Institute, School of Medicine, University of Missouri, Columbia, Missouri, United States

**Keywords:** cornea wound healing, mustard gas, ferroptosis, p38 MAPK signaling, oxidative stress

## Abstract

**Purpose:**

Sulfur mustard gas (SM) exposure to eyes causes multiple corneal injuries including stromal cell loss in vivo. However, mechanisms mediating stromal cell loss/death remains elusive. This study sought to test the novel hypothesis that SM-induced toxicity to human corneal stromal fibroblasts involves ferroptosis mechanism via p38 MAPK signaling.

**Methods:**

New Zealand white rabbit corneas, naïve and SM exposed (200 mg-min/m^3^ for eight minutes and collected after three days) were used to examine the levels of cell death and reactive oxygen species (ROS) for in vivo studies. Donor human corneas were used to generate primary human corneal stromal fibroblasts (hCSF) for in vitro studies. The hCSFs were exposed to nitrogen mustard (NM; SM analogue) at various timepoints (30 minutes, eight hours, and 24 hours). A p38 MAPK specific inhibitor, SB202190, was also used. Quantitative reverse transcription polymerase chain reaction, Western blotting, reactive oxygen species (ROS), lipid peroxidation, live/dead assay, and RNASeq were used in various investigations.

**Results:**

SM caused a significant increase in cell death and ROS production three days after SM exposure in rabbit corneas. NM exposure to hCSF demonstrated a significant increase in ROS, lipid peroxidation, and ferroptosis biomarkers ACSL4 (inducer) and significant decrease in reducer (SLC7A11 and GPX4) compared to controls in a time-dependent manner. The inhibition of p38 MAPK promoted cell survival and reduced ROS production following mustard gas exposure.

**Conclusions:**

The results of in vivo and in vitro investigations uncovered a novel mechanism that mustard gas toxicity to the cornea involves ferroptosis pathway and p38 MAPK activation.

Sulfur mustard (SM), a vesicating and alkylating agent, has been frequently used since World War I and most recently in the Syrian civil war.^1^ SM has profound damage on exposed tissues of the body including the cornea.^2^ Ocular injuries are reported in 90% of victims exposed to SM.^3^ It is reported that ocular SM exposure leads to a time-dependent structural and cellular damage to corneal epithelium, stroma, and endothelium.^4^

An ophthalmic in vivo confocal imaging in live rabbits showed significantly decreased stromal cell density and increased dead cells/debris in the anterior stroma of rabbit eyes exposed to SM, which is likely due to changes in oxidative stress.^5^ Nonetheless, the precise cellular mechanism mediating SM-induced cell loss in stromal fibroblasts/keratocytes remains elusive. SM-induced cell death in the corneal epithelium has been associated with oxidative stress, reactive oxygen species (ROS) generation, and DNA damage.^6^^,^^7^ Corneal epithelium toxicity from the nitrogen mustard (NM), a surrogate for SM, was linked to oxidative stress and expression of 228 unique proteins associated with ferroptosis.^8^ However, the impact of oxidative stress and its potential role in corneal stromal cell death and modulation of ferroptosis in SM exposed cells is largely unknown.

Ferroptosis is a form of cell death that occurs in response to an inflammatory response by the iron-dependent lipid peroxidation.^9^ Iron-dependent lipid peroxidation has been reported to be correlated with excess glutamate and oxidative stress.^10^ Recent publications have shown that oxidative stress is a key element of ferroptosis.^9^ Additionally, involvement of p38 mitogen-activated protein kinase (p38 MAPK) transduction proteins in ferroptosis-associated cellular death processes.^11^ Although the precise mechanism initiating ferroptosis remains elusive, many studies show that mitochondrial function and cell metabolism play an important role in this process.^12^ This study investigated whether ferroptosis and p38 MAPK signaling pathway are part of a regulatory mechanism of mustard gas–induced corneal injury using human cornea in vitro and rabbit cornea in vivo*.*

## Methods

### Animal Care

Male New Zealand white rabbits were used in this study. Rabbits were used in adherence to the ARVO Statement for the Use of Animals in Ophthalmic and Vision Research. The rabbits were purchased from Charles Rivers Laboratories (Wilmington, MA, USA). All arriving rabbits (2.4 to 4.0 kg) showing no signs of ocular disorders were quarantined under veterinarian supervision for two weeks and given certified feed and water as desired. Rabbits were sheltered in rooms with a humidity of 50% ± 20%, temperature of 16°C to 22°C, and 12-hour light/dark cycle each day.^11^

### Cell Culture

Primary human corneal stromal fibroblasts (hCSF) were generated from cadaver corneas acquired from Saving Sight in Kansas City, Missouri, following the reported method.^11^ In brief, the epithelial and endothelial cells from the cornea were removed using a no. 15 surgical blade leaving the stroma intact as reported previously.^11^ The stroma was then cut into eight congruent pieces (buttons) and plated onto a T25 flask with 1 mL medium. Buttons received minimum essential media with 10% fetal bovine serum (FBS) until hCSF became confluent. Cell cultures were incubated and stored at 37°C in 5% CO_2_. All experiments were performed at passage two or three. The hCSFs were cultured with or without NM (100 ng/mL) at various time for the duration of the experiment with or without pretreated of SB202190, a p38 MAPK specific inhibitor, at a concentration of 50 µM (Cat no. 72632; STEMCELL Technologies, Vancouver, BC, Canada). Vehicle treatment (VT) group included equal concentration/volume of vehicle (DMSO for p38 MAPK inhibitor or serum-free media of NM) and collected at the final timepoint of study.

### Mustard Gas Exposure to Rabbit Eye In Vivo and Human Cornea In Vitro

The right eyes of rabbits received SM gas via goggle method (200 mg-min/m^3^ for eight minutes) at MRI Global as published previously.^4^ Contralateral (left) eyes served as naïve controls. The study had two groups: Naive (*n* = 6) and SM (*n* = 6). Three days after initial SM exposure, rabbits were humanely euthanized and corneas were collected, snap-frozen in optimal cutting temperature compound (OCT), stored at −80°C, and serial-sectioned to be used for immunohistochemistry and immunofluorescence studies. For in vitro human cornea studies, hCSF cells were cultured with or without NM (200 ng/mL) and p38 MAPK inhibitor. The desired concentration of NM was prepared by reconstituting mechlorethamine hydrochloride (Cat no. 122564; Sigma-Aldrich, St. Louis, MO, USA) in culture media.

### Terminal Deoxynucleotidyl Transferase dUTP Nick-End Labeling (TUNEL) Assay

Slides mounted with frozen rabbit corneal tissues were subjected to ApopTag TUNEL assay following vendors instructions (Cat no. S7165; Millipore-Sigma, Burlington, MA, USA). Corneal tissue sections were washed with 5% PBS three time for five minutes each and fixed using a 2:1 dilution of ETOH to acetic acid at −20°C for five minutes. After fixation, tissues were washed with 5% PBS two times for five minutes. Thereafter, tissue sections received 15 µL of equilibrium buffer for 10 seconds, followed by 15 µL of terminal deoxynucleotidyl transferase enzyme and incubation at 37°C for one hour. Then, reaction was stopped by submerging slides in stop/wash buffer for 15 seconds followed by incubation at room temperature for 10 minutes. Thereafter, tissue sections received 15 µL of anti-digoxigenin antibody conjugate (rhodamine fluorochrome) and were incubated for 30 minutes and washed with PBS three times (five minutes each time). Tissue sections were mounted with anti-fade 4′,6-diamidino-2-phenylindole (DAPI) (Cat no. H-1200-10; Vector Laboratories, Newark, CA, USA). Sections were fluorescently imaged at 358 ± 20 nm excitation wavelength and 461 ± 20 nm emission wavelength to detect nuclei and fluorescently imaged at 490 ± 20 nm excitation wavelength and 520 ± 20 nm emission wavelength to detect apoptosis.

### Reactive Oxygen Species (ROS) Assay

ROS detection was performed in rabbit corneas in vivo and human corneas in vitro using 2′,7′-dichlorodihydrofluorescein diacetate (H_2_DCFDA) (Cat no. D399; Thermo Fisher Scientific, Waltham, MA, USA) following vendors’ directives. For in vivo experiments*,* slides mounted with rabbit sections were washed in PBS and received 50 µL of H_2_DCFDA diluted to 1:40 in 5% BSA at room temperature for 3 hours. Then, tissue sections were washed with PBS and fixed in antifade DAPI medium (VECTASHEILD) and mounted with a glass coverslip. Sections were fluorescently imaged at 358 ± 20 nm excitation wavelength and 461 ± 20 nm emission wavelength to detect nuclei, and fluorescently imaged at 488 ± 20 nm excitation wavelength and 510 ± 20 nm emission wavelength to detect ROS. For in vitro experiments, hCSFs cultures grown in 96 well with vehicle, NM, p38 MAPK inhibitor, NM+p38 MAPK inhibitor, were used. At selected end points, cultures received 100 µL of H_2_DCFDA diluted to 2.5:1000 in serum-free FluoroBrite DMEM culture media (Cat no. A1896701; Thermo Fisher Scientific). After mixing, the 96-well plates were incubated at 37°C in 5% CO2 for 30 minutes. Fluorescent measurements were recorded using a microplate reader at 493 ± 20 nm excitation wavelength and 523 ± 20 nm emission wavelength.

### Lipid Peroxidation (LPO) Assay

Lipid peroxidation assay was performed in rabbit cornea in vivo and human cornea in vitro using a Click-iT Linoleamide Alkyne (LAA) kit (Cat no. C10446, C10447; Thermo Fisher Scientific). LAA solution at a concentration of 50 µM was added to hCSFs before and during NM treatments at 30 minutes, eight hours, and 24 hours. At end points, cells were washed three times with PBS containing Tween-20 and blocked with 1% BSA. Click-iT reaction cocktail was subsequently added according to manufacturer's instructions for 30 minutes. Cells were then washed with PBS and fixed in antifade DAPI medium and mounted with a glass slide coverslip. Cells were fluorescently imaged at 358 ± 20 nm excitation wavelength and 461 ± 20 nm emission wavelength to detect nuclei, and fluorescently imaged at 488 ± 20 nm excitation wavelength and 510 ± 20 nm emission wavelength to detect lipid peroxide products.

### Quantitative RT-PCR (qRT-PCR)

The total RNA was isolated using the RNeasy kit (Cat no. 74636; Qiagen, Valencia, CA) and converted to cDNA using the GoScript Reverse Transcription System (Cat no. A5001; Promega Corporation, Madison, WI, USA) following manufacture's protocol from hCSFs grown in ±NM at various times.^13^ The relative mRNA expression was calculated using the 2-ΔΔCt method and a relative fold change over the corresponding control values following methods reported previously.^13^ The qRT-PCR was performed in triplicate for each sample, and a minimum of three independent experiments were conducted. The primer sequences with accession number are provided in the Table.

**Table.** List of Primer Names, Accession Numbers, and Sequences for qRT-PCR

S. No.	Gene Name	Accession No.	Forward Sequences (5′–3′)	Reverse Sequences (3′–5′)
1	GAPDH	NM_002046.3	GACCTGACTGACTACCTCAT	ATGTCACGCACGATTTCC
2	ACSL4	NM_004458	CTTGCCTTTGGCTCATGT	GGACTGGTCAGAGAGTGTAA
3	SLC7A11	NM_014331	GAGAAAGTGCAGCTGAAGAG	GCCCTTAGGAGAGATGAAGA
4	GPX4	NM_002085	CGGGCTACAACGTCAAAT	CCTTGGGTTGGATCTTCATC
5	p53	NM_001276761	AGGTTGGCTCTGACTGTA	GTGTGATGATGGTGAGGATG
6	MAP2Ks	NM_030662.4	GAAAGAGGCCAAGAGGATTC	AAGCACCAGATCATGCAC
7	CHOP	NM_001195054.1	ACTCTTGACCCTGCTTCT	TCTGACTGGAATCTGGAGAG
8	p38	XM_002717471.3	CACGATCCTGATGATGAACC	CCCTGCTTTCAAAGGACT
9	CAS3	NM_004346	CCACAGCACCTGGTTATT	AAGCTTGTCGGCATACTG
10	CAS9	NM_001229	CGAACTAACAGGCAAGCA	GTCTGAGAACCTCTGGTTTG

### Western Blotting

Total protein lysates from hCSFs grown in vehicle, NM, p38 MAPK inhibitor or NM+p38 MAPK inhibitor were prepared using RIPA lysis buffer (Cat no. sc-24948; Santa Cruz Biotechnology, Dallas, TX, USA) and protein concentrations were quantified using Bradford assay (Cat no. 500006; Bio Rad Labs, Hercules, CA, USA) following reported methods.^13^ Western blotting was performed using antibodies specific for ɑSMA (Cat no. M0851; DAKO-Agilent, Santa Clara, CA, USA), p38 MAPK (Cat no. Sc-271120; Santa Cruz Biotechnology), and pp38 MAPK (Cat no. Sc-7973; Santa Cruz Biotechnology) as published previously.^14^ In brief, primary and secondary antibodies were used at 1:100 and 1:500 dilutions, respectively. The signals on membranes were activated with luminol to peroxide buffer (1:1) using SuperSignal West Dura Extended Duration Substrate (Cat no. 34075; Thermo Fisher Scientific) and recorded with iBright imager (Cat no. A44114; Thermo Fisher Scientific).

### LIVE/DEAD Cell Assay

Real-time apoptotic and necrotic cell death was determined by measuring outer leaflet-bound phosphatidylserine with HCS LIVE/DEAD Green Kit (Cat no. H10290; Thermo Fisher Scientific) following vendor's protocol. In brief, in a 96-well plate, each well was seeded with 3 × 10^3^ hCSFs using 200 µL of culture media supplemented with 10% FBS until 70% confluency. Thereafter, cultures were serum starved for 24 hours then received NM (100 µM) or vehicle and incubated for 30 minutes, eight hours, or 24 hours. Fluorescent measurements were recorded using a microplate reader at 485 ± 20 nm excitation wavelength and 523 ± 20 nm emission wavelength.

## Results

### Mustard Gas Exposure Increase Cell Death and Oxidative Stress in Rabbit Cornea

**Figure 1.**
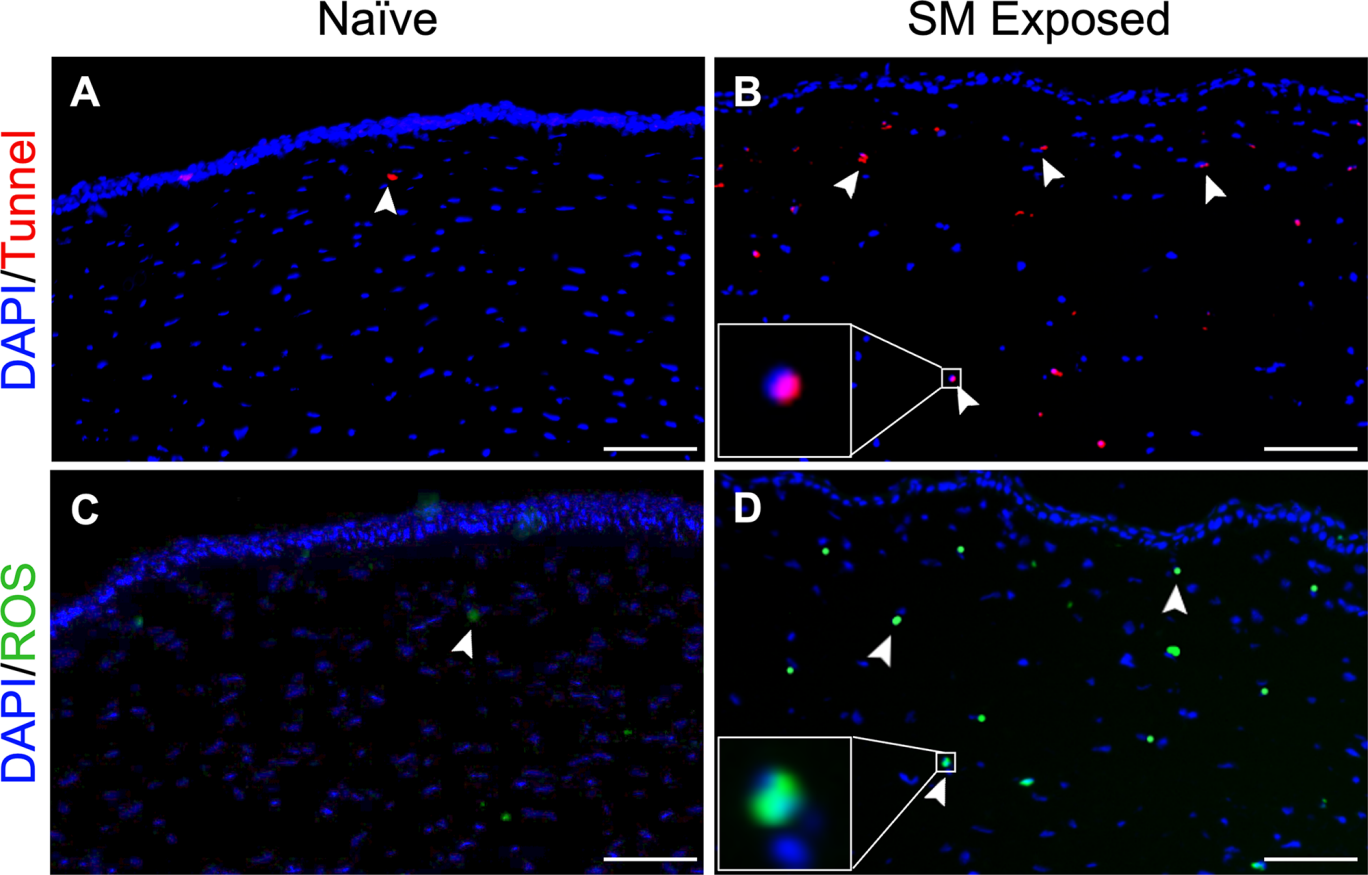
Rabbit corneas exposed to mustard gas had increased cell death and ROS production. Rabbit eyes were exposed to sulfur mustard and after three days were stained for cell death (**B**) and oxidative stress (**D**). SM exposed corneas had a significant increase in tunnel positive staining at day 3 (**B**) compared to naïve group (**A**). Tunnel-positive cells are red (*white arrow*). H_2_DCFDA was used to identify cells with excess ROS production in SM-exposed corneas. SM exposure has increased ROS (*green*) staining in the corneal stroma (**D**) compared to naïve group (**C**). Data shown represent three independent experiments (*n* = 3). Representative immunohistochemistry images show Tunnel(*red*), ROS (*green*), and DAPI (*blue*) at three days after SM exposure. *Scale bar*: 100 µm.

Rabbit naïve and SM exposed corneas were tested for the cell death and oxidative stress three days post SM. A significantly increased TUNEL+ cells in stroma were observed in SM-exposed eye (Fig. 1B; *P* < 0.01) compared to the naïve (Fig. 1A). Furthermore, the SM exposed corneas showed significantly enhanced ROS levels in stroma (Fig. 1D; *P* < 0.01) compared to the naïve (Fig. 1C). Collectively, this data suggested that oxidative stress contributes to stromal cell death in cornea post SM exposure.

### Mustard Gas Exposure Increases Lipid Peroxidation

**Figure 2.**
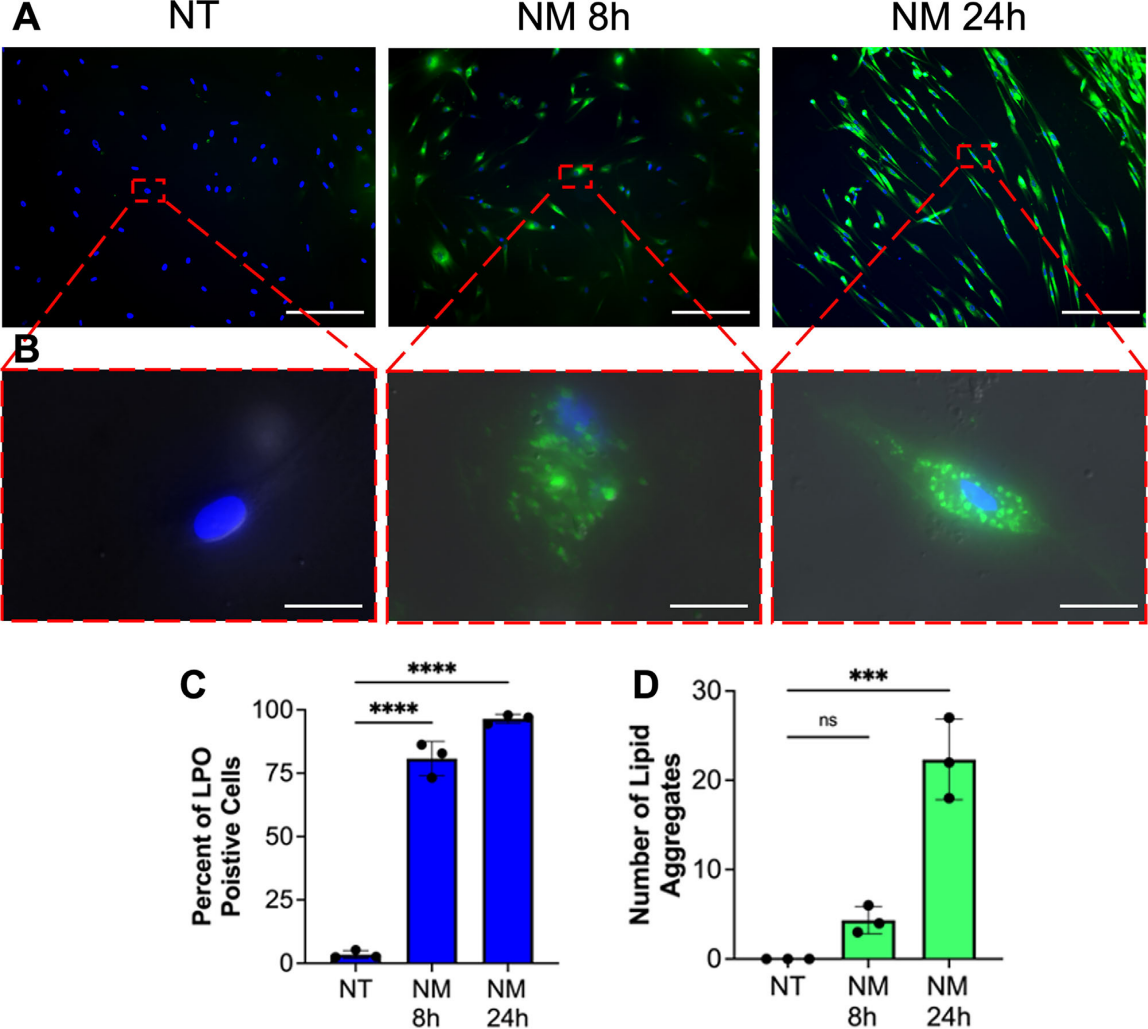
Mustard gas exposure results in increase lipid peroxidation in vitro hCSFs were exposed to nitrogen mustard were accessed for lipid peroxidation. Click-iT LAA was used to detect lipid peroxidation (**A**) and LPO byproduct aggregates within a single cell (**B**). The ratio of lipid peroxide positive cells to total cells was measured (**C**). A significant increase in the ratio of cells with lipid peroxidation byproducts was detected at eight hours (*P* < 0.0001) and 24 hours (*P* < 0.0001). Furthermore, the number of LPO byproduct aggregates was quantified (**D**). A significant increase in the amount of lipid peroxidation byproducts was present at 24 hours (*P* < 0.0001) compared to the no-treatment (NT) group. Data shown is from six primary cultures per group with samples tested in triplicates. Representative immunocytochemistry images show lipid peroxide byproducts (*green*), and DAPI (*blue*) at NT, eight hours, and 24 hours. *Error bars* depict standard deviation. * *P* < 0.05, *** *P* < 0.001, and **** *P* < 0.0001. *Scale bar A*: 100 µm; *Scale bar B*: 20 µm

LPO is directly related and is a key component of the oxidative stress. In a controlled cell culture environment, hCSFs exposed to NM, a SM surrogate, caused significantly increased LPO levels in a time-dependent manner at eight hours (*P* < 0.0001) and 24 hours (*P* < 0.0001) compared to NM non-treated control (Figs. 2A–C). An evaluation of fluorescence in single cell revealed the presence of LPO by-product aggregates forming in the cytoplasm of hCSF at eight hours and 24 hours (Fig. 1B). The number of LOP byproduct aggregates increased at eight hours, but a significant increase occurred at 24 hours after NM (*P* < 0.001) compared to the no-treatment group (Fig. 2D).

### Mustard Gas Exposure Induces Ferroptosis

**Figure 3.**
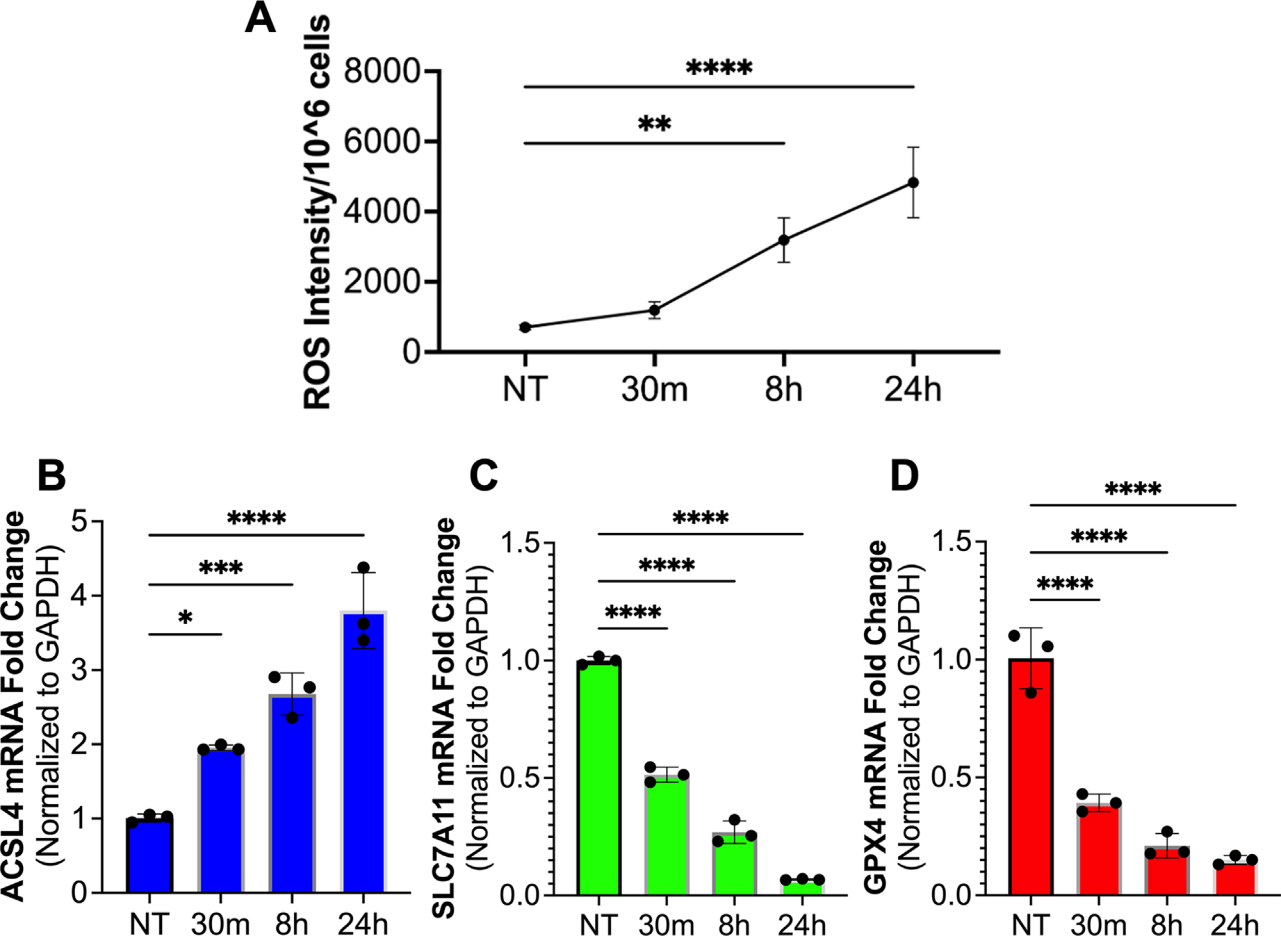
Mustard gas activates ferroptosis pathway in hCSF. The hCSF were exposed to nitrogen mustard, a sulfur mustard analogue, and accessed for ferroptosis-mediated cell death. H_2_DCFDA was used to detect total ROS production in hCSF exposed to mustard gas. A significant increase in ROS (**A**) was detected at eight hours (*P* < 0.01) and 24 hours (*P* < 0.001) compared to NT. The qRT-PCR was used to access ferroptosis markers including ACSL4 (**B**), SLC7A11 (**C**), and GPX4 (**D**). ACSL4 initiates lipid peroxidation while SLC7A11 and GPX mitigate excess ROS production from lipid peroxidation. A significant increase in ACSL4 at 30 minutes (*P* < 0.05), eight hours (*P* < 0.001), and 24 hours (*P* < 0.0001) was detected. Conversely, a significant decrease in SLC7A11 and GPX4 was detected at 30 minutes (*P* < 0.0001), eight hours (*P* < 0.0001), and 24 hours (*P* < 0.0001). Data shown is from six primary cultures per group with samples tested in triplicates. *Error bars* depict standard deviation. * *P* < 0.05; *** *P* < 0.001; **** *P* < 0.0001

The hCSFs were exposed to nitrogen mustard to identify oxidative stress induced ferroptosis cell death by measuring ROS, ACSL4, SLC7A11, and GPX4 expression at 30 minutes, eight hours, and 24 hours after NM. Mustard gas induced a significant time-dependent increase in ROS production in hCSF compared to the no-treatment group (Fig. 3A; *P* < 0.01 or *P* < 0.001). Additionally, the ACSL4 levels were significantly increased in a time-dependent manner compared to the no-treatment group (Fig. 3B; *P* < 0.05, *P* < 0.001, or *P* < 0.0001) indicating the induction of ferroptosis cell death pathway. Moreover, a significant time-dependent decrease in expression of LPO reducers, SLC7A11 (Fig. 3C) and GPX4 (Fig. 3D), occurred in hCSFs after mustard gas exposure compared to the no-treatment group (Figs. 3C–D, *P* < 0.0001). This data indicated that mustard gas induces ferroptosis in hCSFs.

### Upregulation of p38 MAPK Signaling During Mustard Gas Induced Ferroptosis

**Figure 4.**
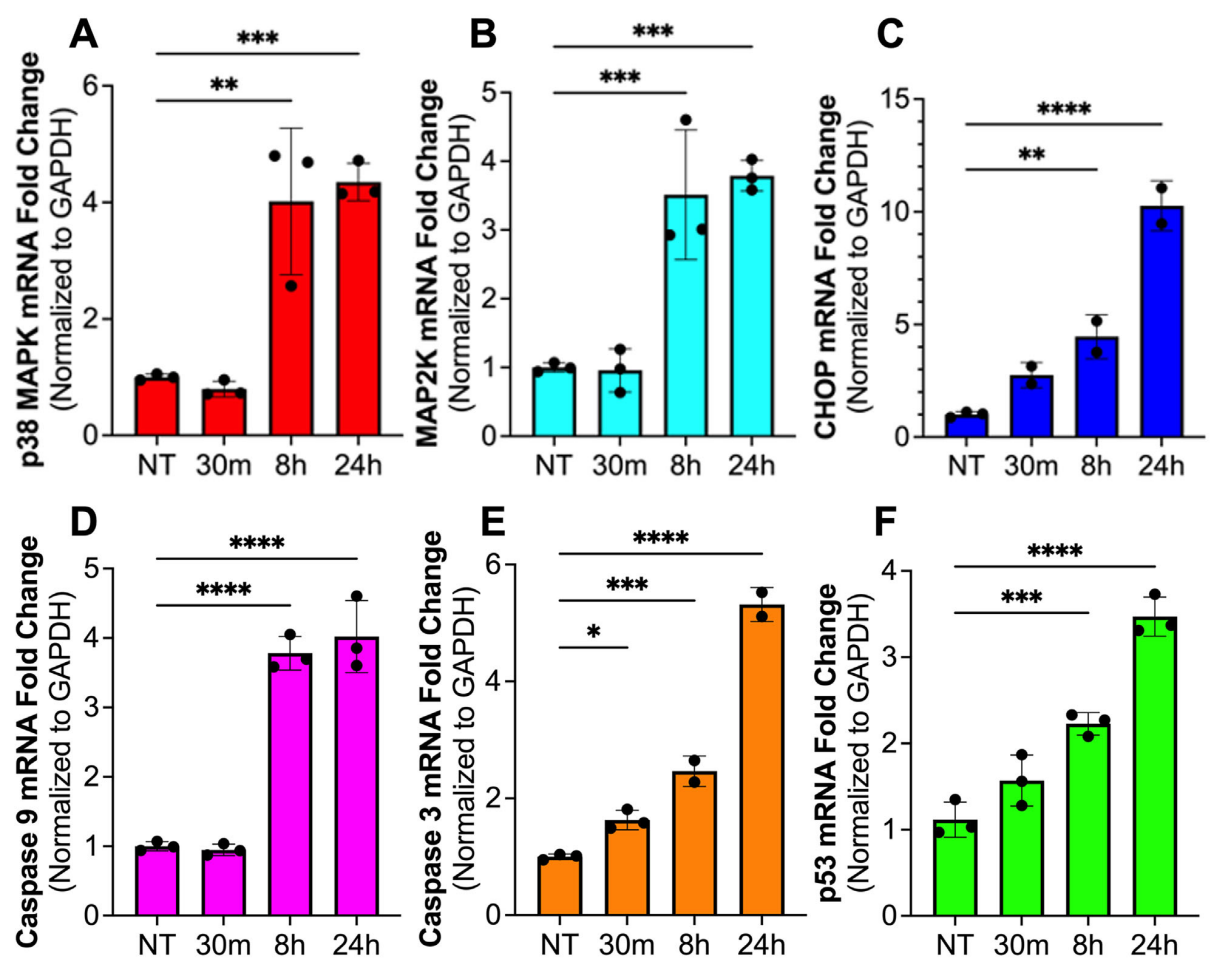
SM-induced hCSF ferroptosis activates p38 MAPK signaling. The hCSF exposed to mustard gas were accessed using qRT-PCR for p38 MAPK signaling. A significant increase in p38 MAPK mRNA transcription (**A**) was detected at eight hours (*P* < 0.01) and 24 hours (*P* < 0.001). MAP2Ks (**B**) that phosphorylate p38 MAPK increased at eight hours (*P* < 0.001) and 24 hours (*P* < 0.001). CHOP (**C**) a target of p38 MAPK increased at eight hours (*P* < 0.01) and 24 hours (*P* < 0.0001). An increase in caspase 9 (**D**) was detected at eight hours (*P* < 0.0001) and 24 hours (*P* < 0.0001) with a corresponding increase in caspase 3 (**E**) at 30 minutes (*P* < 0.05), eight hours (*P* < 0.001), and 24 hours (*P* < 0.0001). Finally, p53 (**F**) increased at 30 minutes (*P* < 0.001) and 24 hours (*P* < 0.0001). Data shown is from six primary cultures per group with samples tested in triplicates. *Error bars* depict standard deviation. * *P* < 0.05; ** *P* < 0.05; *** *P* < 0.001; **** *P* < 0.0001

An involvement of p38 MAPK signaling in mustard gas induced ferroptosis in cornea through oxidative stress was examined by measuring components of the p38 MAPK pathway, ROS, and inflammasome-mediated caspases. The mRNA levels of p38 MAPK (Fig. 4A), mitogen-activated protein kinase kinases, MAP2K (Fig. 4B), and C/EBP homologous protein (CHOP) (Fig. 4C) had a significant increase at eight hours, and 24 hours in hCSF exposed to mustard gas compared to the no-treatment group (Figs. 3A–C; *P* < 0.01, *P* < 0.001, or *P* < 0.0001). MAP2K is a dual-specificity kinase enzyme which phosphorylates p38 MAPK to activate signaling. This increase correlates with a significant increase in total cell ROS production (Fig. 1D). Furthermore, inflammasome-mediated caspase 9 (Fig. 4D) and caspase 3 (Fig. 4E) were significantly enhanced in a time-dependent manner after mustard gas exposure compared to the no-treatment control group (Figs. 3D, 3E; *P* < 0.05, *P* < 0.001, or *P* < 0.0001). In addition, p53 significantly increased in a time-dependent manner (Fig. 4F; *P* < 0.001 or *P* < 0.0001), which has been shown to correlate with ferroptosis pathway.^10^ These data together exhibited that p38 MAPK is activated during SM-induced ferroptosis.

### The p38 MAPK Activity Correlates With Ferroptosis

**Figure 5.**
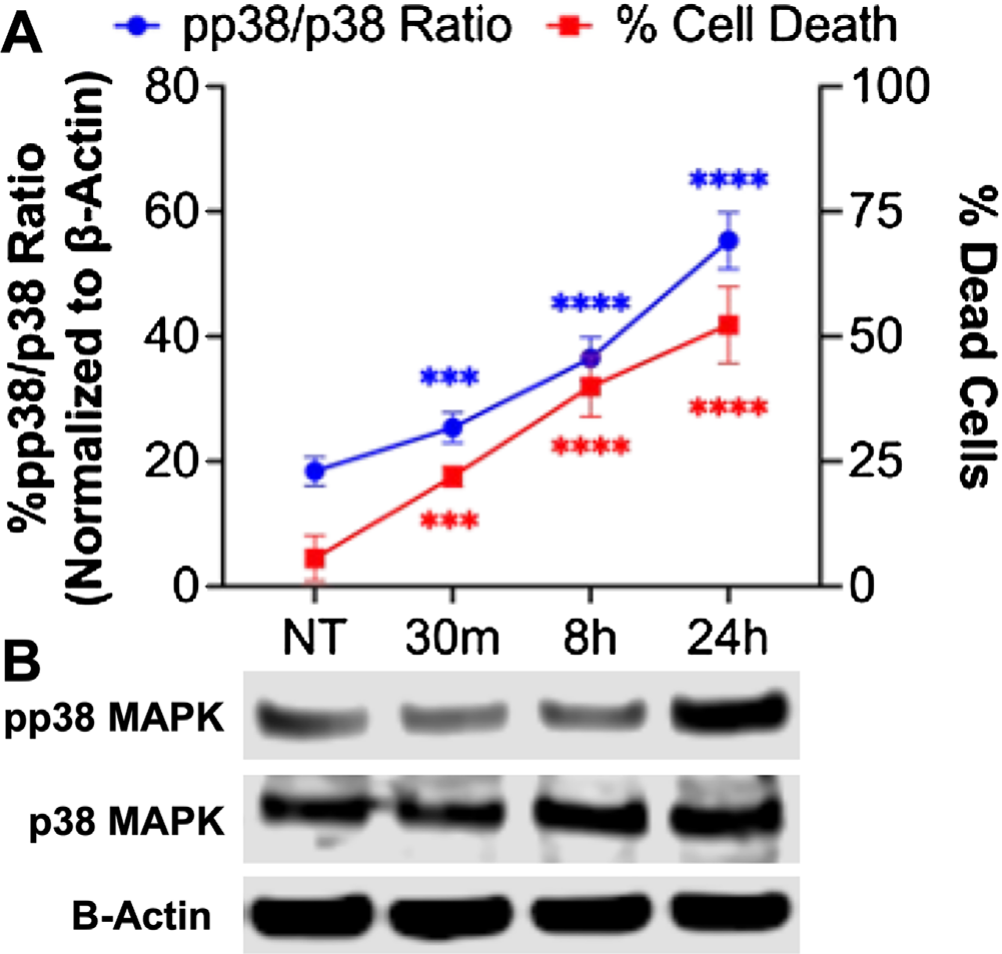
The p38 MAPK activation correlates with mustard gas induced ferroptosis. hCSFs exposed to mustard gas were subjected to live/dead assay for cell death and to western blotting to test phosphorylated p38 MAPK to p38 MAPK ratio (pp38/p38). Cell death (**A**) significantly increased at 30 minutes (*P* < 0.001), eight hours (*P* < 0.0001), and 24 hours (*P* < 0.0001). Panel **B** shows representative Western blot images. The pp38/p38 MAPK ratio significantly increased at 30 minutes (*P* < 0.001), eight hours (*P* < 0.0001), and 24 hours (*P* < 0.0001). The p38 MAPK activation had a direct time-dependent increase with ferroptosis in hCSF. Data shown is from six primary cultures per group with samples tested in triplicates. *Error bars* depict standard deviation. *** *P* < 0.001; **** *P* < 0.0001

**Figure 6.**
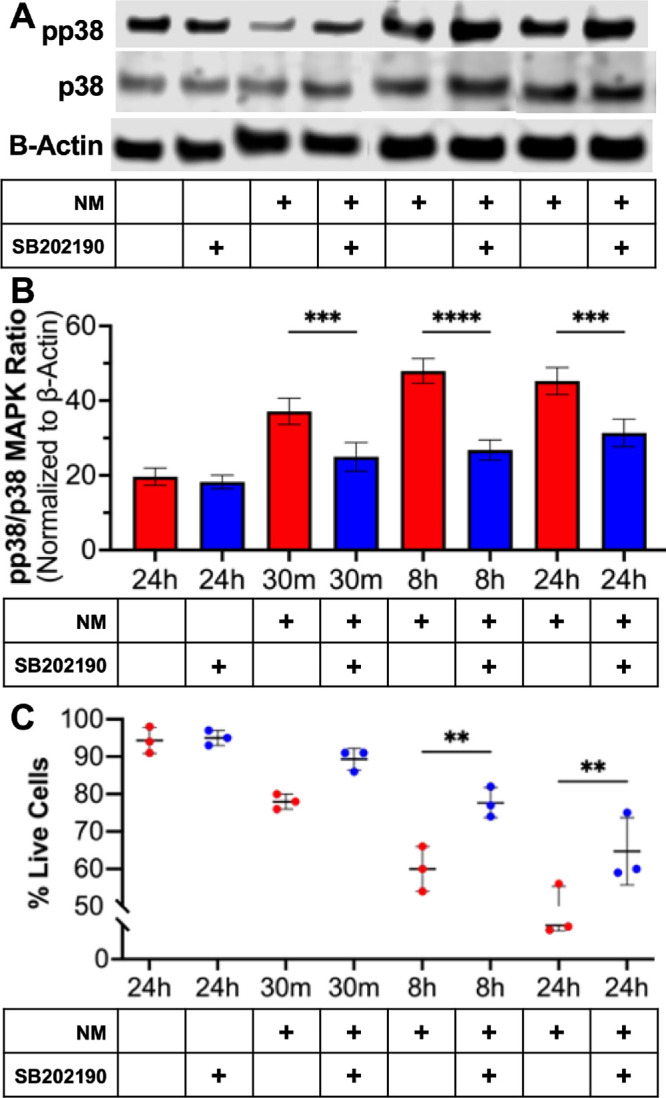
Inhibiting p38 MAPK prevented mustard gas induced hCSF ferroptosis. The hCSF were pretreated with or without SB202190 to inhibit p38 MAPK activity then exposed to mustard gas. (**A**) Representative Western blot images. SB202190 significantly inhibited p38 MAPK phosphorylation (**B**) at 30 minutes (*P* < 0.001), eight hours (*P* < 0.0001), and 24 hours (*P* < 0.0001). The inhibition of pp38 MAPK in hCSF significantly promoted cell viability (**C**) after mustard gas exposure at eight hours (*P* < 0.01) and 24 hours (*P* < 0.01). The addition of (+) shows treatment added to culture media. Data shown is from three primary cultures per group with samples tested in triplicates. *Error bars* depict standard deviation. (*Blue Bars* = addition of SB202190 and *Red* = without SB202190.) (** *P* < 0.05; *** *P* < 0.001; **** *P* < 0.0001)

**Figure 7.**
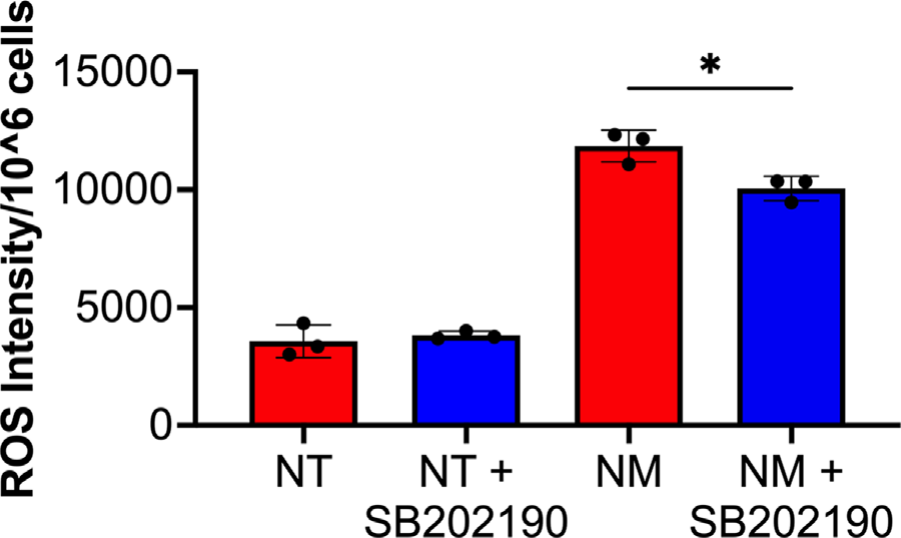
Inhibition of p38 MAPK in hCSF significantly reduced mustard gas–induced ROS production. The hCSFs were pretreated with or without SB202190 to inhibit p38 MAPK activity then exposed to mustard gas for 24 hours. The inhibition of pp38 MAPK in hCSF significantly reduced ROS production after mustard gas exposure (*P* < 0.05). Data shown is from three primary cultures per group with samples tested in triplicates. *Error bars* depict standard deviation. * *P* < 0.05

The ratio of phosphorylated-p38 MAPK (pp38) to p38 MAPK proteins and its inhibition with SB202190 in conjunction of cell death was assessed to determine p38 MAPK activity and its impact on ferroptosis (Figs. 5^6^–7). The pp38/p38 MAPK levels significantly increased (Fig. 5B, p < 0.001 or p < 0.0001) at a corresponding rate to mustard gas induced hCSF ferroptosis (Fig. 5A; *P* < 0.001 or *P* < 0.0001). In addition, the inhibition of p38 MAPK with SB202190 significantly reduced the expression of p38 MAPK at 30 minutes (*P* < 0.001), eight hours (*P* < 0.0001), and 24 hours (*P* < 0.0001) (Figs. 6A, 6B). The inhibition of p38 MAPK significantly increased the number of live cells after mustard gas exposure at eight hours (*P* < 0.01) and 24 hours (*P* < 0.01) (Fig. 6C). Finally, the inhibition of p38 MAPK resulting in reduced ROS production in hCSF after mustard gas exhibited its regulatory role in mustard gas–induced corneal injury (Fig. 7).

## Discussion

SM gas, unlike other warfare agents, causes instant blindness and severe injury to the cornea upon contact to eyes.^14^ SM ocular exposure has been shown to cause multiple corneal pathologies including corneal edema, inflammation, fibrosis/haze, neovascularization, and ulcer and are collectively referred to as mustard gas keratopathy (MGK).^15^ Despite recent progress in the pathogenesis of MGK, the precise understanding of mechanisms of SM toxicity to cornea is poorly understood.^16^ The present study identifies a novel mechanism contributing to the loss of stromal fibroblast/keratocyte cell density in cornea after SM exposure. Stromal keratocytes and fibroblasts are primarily responsible for maintaining transparency and tissue repair in an injured cornea in coordination with other cells.^17^^–^^19^

Rabbit corneas showed a significant increase in dead cells and ROS in the anterior stroma compared to naïve corneas post SM exposure (Fig. 1). Additionally, in vitro experiments with human corneal stromal fibroblasts demonstrated a time-dependent increase in ROS (Fig. 1) and LPO (Fig. 2), and alterations in ferroptosis markers, ACSL4, SLC7A11, and GPX4 (Fig. 3). This observation supported the hypothesis that SM-induced corneal stromal cell loss involves ferroptosis. Nonocular literature supports our hypothesis because ferroptosis has been implicated in cell death after high ROS production during inflammatory states.^9^

Apart from increased ROS from iron metabolism, ferroptosis is also involves the accumulation of lipid peroxide by-products in cytoplasm of the cells. In current study, the hCSF cells exposed to mustard gas exhibited significantly increased level of LPO and formation of lipid peroxide by-products aggregates in a time-dependent manner (Fig. 2). At eight hours and 24 hours the ratio of LPO+ cells was similar; however, the number of lipid peroxide byproducts aggregates was significantly higher at the latter timepoint, 24 hours. The aggregates appear as “holes” in the cell membrane formed because of excessive levels of LPO and are generally seen at the terminal stages of ferroptosis. A detection of significant increase in LOP byproducts at 24 hours in hCSFs with increased cell death corroborates with noncorneal literature.^20^ The discovery of ferroptosis-induced cell death after SM in conjunction with increased lipid peroxidation presents a novel mechanism in mustard gas toxicity to the cornea and offers an attractive target for developing newer mitigation approaches.

Mechanistically, ferroptosis is controlled by proteins that mitigate or exacerbate either ROS or lipid peroxide by-products. The ACSL4, SLC7A11, and GPX4 proteins serve an important role in ferroptosis. Thereby, these protein regulators were evaluated in hCSFs in the presence and the absence of NM. The increase in ACSL4 expression was significant and time-dependent in vitro (Fig. 3) which aligns with our previous^5^ and ongoing studies finding the role of oxidative stress in MGK. ACSL4 is a correlative marker for ferroptosis and is shown to be involved in esterification of arachidonic acid and oxidation of lipids.^20^ On the other hand, detection of the significantly decreased SLC7A11 and GPX4 proteins in a time-dependent in hCSFs exposed to nitrogen mustard further revealed presence of ferroptosis (Fig. 3). Both, SLC7A11 and GPX4 proteins, are negative regulators of ferroptosis. SLC7A11 serves to limit ROS production through the increased formation of GSH.^21^ Similarly, GPX4 mitigates ferroptosis by reducing lipid peroxides to nontoxic lipid alcohols.^22^^,^^23^ Both, SLC7A11 and GPX4, in cellular homeostasis are typically upregulated during lipid peroxidation to counterbalance excessive ROS production.^24^ The time-dependent increase in ACSL4 after SM led to the postulate and investigation of the potential involvement of p38 MAPK pathway.

MAPK signaling has been shown to regulate several key ferroptosis proteins targeting both lipid peroxidation and iron regulation. In cancer studies, ACSL4 was found to be induced by MAPK pathway leading to the accumulation of lipid peroxides^25^ and its inhibition led to decreased level of SLC7A11.^26^^,^^27^ Given inhibition of p38 MAPK reduces ferroptosis we postulate that it does this by regulating ACSL4 and SLC7A11 balance. The data from the present study suggests that p38 MAPK signaling promotes ACSL4 protein causing excessive lipid peroxidation and ultimately ferroptosis. Apart from the p38 MAPK signaling, inhibition of JNK pathway in cancer studies is also documented which was not tested in present study.^28^

A significant time-dependent increase in p38 MAPK activity and protein levels were observed in NM exposed hCSF samples (Fig. 5). This analysis suggested that p38 MAPK phosphorylation plays a role in mustard gas induced ferroptosis. The involvement of p38 MAPK in mustard gas activation of ferroptosis is further confirmed by the upregulation of CHOP and p53 by p38 MAPK. The p53 has been associated with activation of ferroptosis in hepatocarcinoma and keratinocytes.^29^^,^^30^ Also, p53 activation is shown to reduce SLC7A11 protein expression.^31^ To further confirm the p38 activity, SB202190 (a specific inhibitor) was implemented. Inhibition of p38 MAPK in hCSF significantly promoted cell survival (Fig. 6C) and reduced ROS production (Fig. 7). This finding demonstrated that p38 MAPK is directly involved in regulating ferroptosis. Given that p38 MAPK signaling regulates multiple cellular functions including cellular growth, proliferation, and metabolism,^32^ further studies are required to study the downstream pathways of p38 MAPK signaling in the context of mustard gas induced corneal injury and MGK. Our ongoing research will be investigating the role of downstream pathways including regulation of iron and ACSL4 to inhibit ferroptosis.

The involvement of various cell death genes in SM induced corneal toxicity and MGK was also revealed by the enrichment of 207 cell death genes (Supplementary Table S1) and protein-protein interaction network (Supplementary Fig. S1A) via RNA-seq analysis of naïve and SM-exposed rabbit corneas collected four weeks after SM. The transcriptomic profiling finding significant alterations in cell death marker genes including CASP1, GSDMD, IL18BP, IL1B, TNF, FAS, RIPK1, and TFRC (Supplementary Fig. S1B), ACSL4, SLC7A11, and GPX4 (Fig. 3), and caspase-3 and -9 (Fig. 4) suggested the role of nonapoptotic (ferroptosis, pyroptosis, and necroptosis) and apoptotic cell death. This is likely due to the SM's ability to directly damage DNA by alkylation, which prevents cell division and prompts cell suicide in affected corneal cells. Unquestionably, detection of all forms of cell death from SM warrants additional studies to uncover precise role of specific cell death in MGK pathogenesis.

In conclusion, the results of the present study show involvement of ferroptosis and p38 MAPK signaling activity in mustard gas–induced corneal injury and MGK pathogenesis. Furthermore, molecular events associated with p38 MAPK and ferroptosis may potentially offer novel targets for developing medical countermeasures to mitigate mustard gas–induced cornea blindness.

## Supplementary Material

Supplement 1

Supplement 2
