# Supplementary material for: Mustard Gas Induced Corneal Injury Involves Ferroptosis and p38 MAPK Signaling
Source: Invest Ophthalmol Vis Sci. 2025 Jan 10;66(1):23. doi: 10.1167/iovs.66.1.23 (PMC11730948; doi:10.1167/iovs.66.1.23)
Supplement: Supplement 1 [file iovs-66-1-23_s001.pdf]

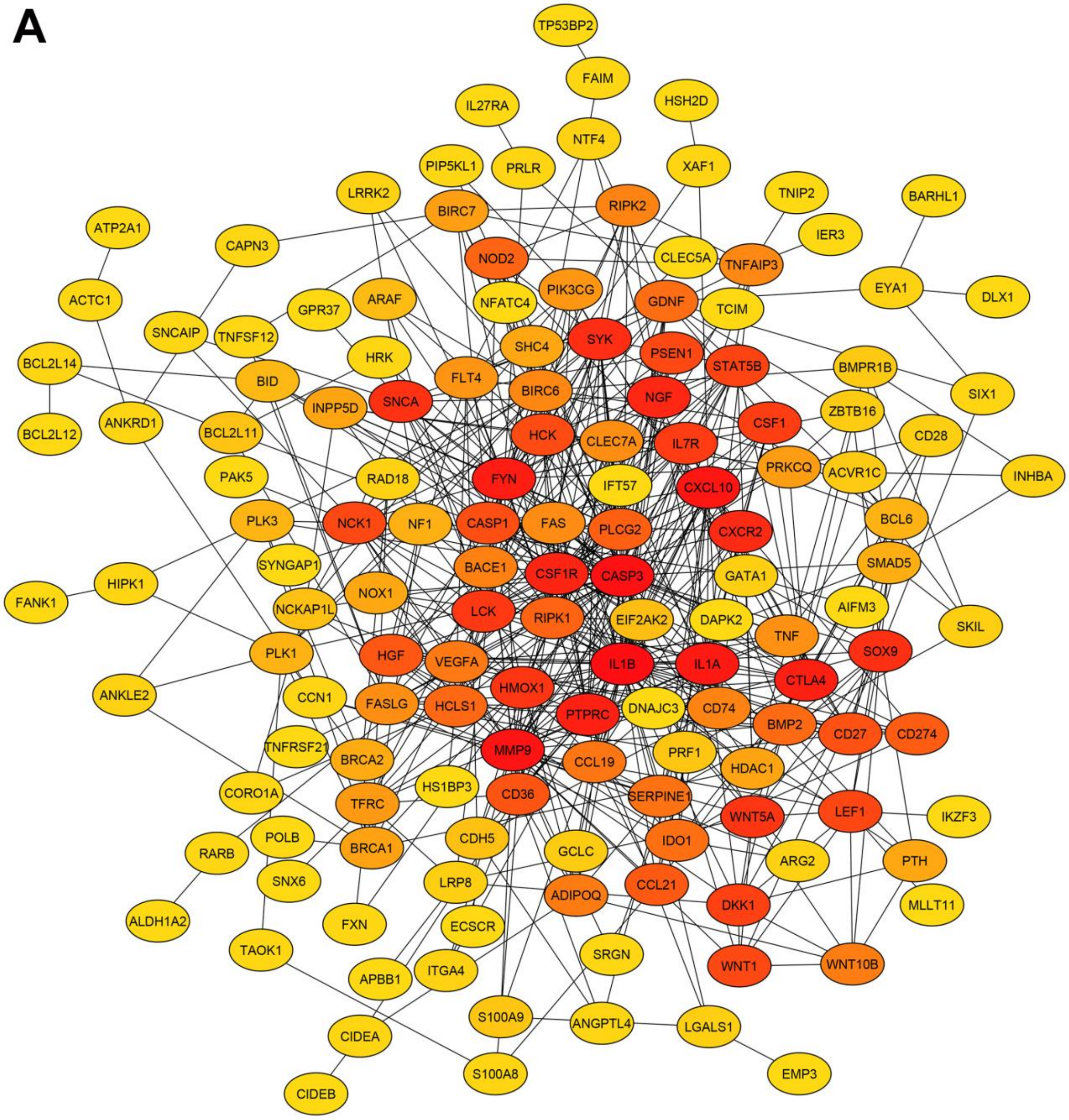

Figure S1A: **Protein-Protein Interaction network of enriched genes involved in the cell death pathway in SM-injured versus naïve rabbit corneas**. The network was generated using Cytoscape and the cytoHubba plugin was used to identify the influential nodes within the network. Nodes are colored based on their influence, with red indicating highly influential node that play a critical role within the network. Edges represent interactions between proteins, that

emphasize the interconnectedness of the nodes implicated in cell death processes following mustard gas exposure.

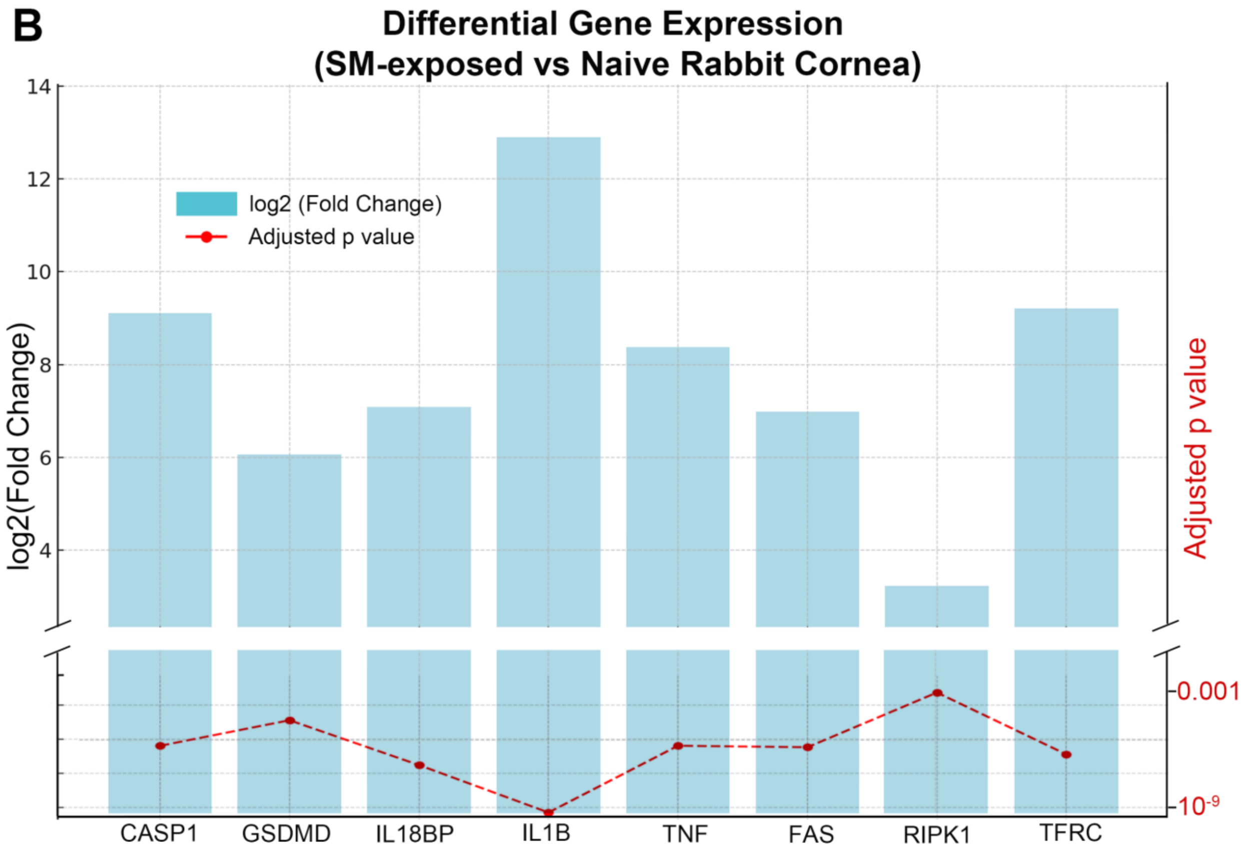

Figure S1B: Cell death markers exhibit significant upregulation in SM-Injured rabbit corneas compared to naïve controls. The log2(Fold Change) (bar plot) and Adjusted p values (line plot) of CASP1, GSDMD, IL18BP, IL1B, TNF, FAS, RIPK1, and TFRC (cell death pathway related genes) are shown. The red line indicates the corresponding adjusted p-values for each gene, plotted on the secondary y-axis. The adjusted p-values used in this study was Benjamini-Hochberg (BH) procedure for controlling the false discovery rate. This adjustment corrects for multiple testing and reduces the likelihood of false positives while identifying differentially expressed genes.

Supplementary Table S1: List of 207 genes enriched in the cell death pathway for SM-injured versus naïve rabbit corneas. It includes log2(Fold Change) representing the relative expression changes, Standard Error (StdErr) of the log2(Fold Change) values, Wald-Statistics (Wald-Stats) for statistical evaluation, p-values that indicate the statistical significance of differential gene expression, and Adjusted p values (p-adj.) corrected for multiple comparisons.
